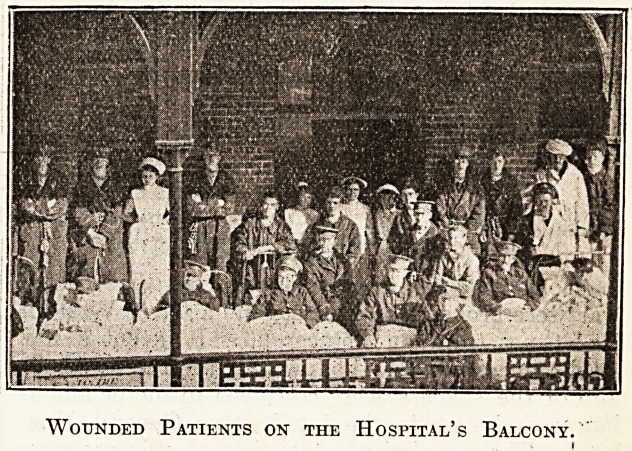# The Naval Red Cross Train in London: Great Northern Central Hospital

**Published:** 1914-11-07

**Authors:** 


### GREAT NORTHERN CENTRAL HOSPITAL.

This institution in the Holloway Road is a good example of the way in which the voluntary hospitals have tried to fulfil their duty to the civil population while at the same time affording treatment to the sick and wounded soldiers. The committee placed 100 beds at the Government's disposal, and already one ward has been occupied by men from the Front. The more convalescent are shown on the balcony in the accompanying photograph. The existing accommodation was increased by a large number of emergency beds before any offer was made for the reception of the soldiers. The strain of the double work on Mr. Cuthbert Panter, the secretary, and the staff generally has been very great, but they are cheered in the work, not only by consciousness of its value, but also by the belief that the hospital's supporters will show by their prompt assistance that they appreciate and endorse what has been done.

Wounded Patients on the Hospital's Balcony.

## Figures and Tables

**Figure f1:**